# Supplementary material for: A metabolomics study of ascorbic acid‐induced in situ freezing tolerance in spinach (Spinacia oleracea L.)
Source: Plant Direct. 2020 Feb 24;4(2):e00202. doi: 10.1002/pld3.202 (PMC7036623; doi:10.1002/pld3.202)
Supplement: Supplementary file 2 [file PLD3-4-e00202-s002.pdf]

Dear Editor:

We appreciate all reviewers for their valuable time and comments for our manuscript titled "**A metabolomics study of ascorbic acid-induced *in situ* freezing tolerance in spinach (*Spinacia oleracea* L.)**".

Reviewer #1 and #2 did not require any revisions but Reviewer# 3 did. We have done our best to revise the manuscript based as per reviewer 3's comments, which involved new metabolite analysis using four biological replications, each with triplicates as described below.

Response to Reviewer #3

The major comment from reviewer #3 was that replication number was too low in the metabolite profiling experiment, i.e. 2 biological replications each with 2 technical replications. In order to resolve this problem, we newly performed metabolite profiling experiment, using **4 biological replications per biological condition, each with 3 technical replications, resulting in a total of 12 replications per biological condition**. Please also note that for the revised version we did not use any of the metabolite data from the original submission. This was recommended by the ISU metabolomics center's technicians/manager since the GC-MS instrumentation used for the original analysis had undergone some servicing (new filament, new column, clean source etc.) in the last few months. They felt that this so-called 'renewal' of the instrument could potentially result in 'non-biological' discrepancy in the data when compared to the original one. Therefore, we decided to use only newly obtained data from 4 biological replications, each with triplicate samples.

While absolute concentrations of metabolites for the most part were generally lower in the newly obtained data, general trend of relative changes in individual metabolites were almost identical to the original data for the two biological conditions, i.e. AsA-treated vs. F-control. Below, is a tabulated summary of the major changes noted between the new (revised version) and the previous (original submission) metabolite data:

<table>
<tr><th>Original submission</th><th>Revised version</th></tr>
<tr><td>47 metabolites identified</td><td>46 metabolites identified
‘Homoserine’ not detected in new analysis</td></tr>
<tr><td>The abundance of 22 metabolites was significantly different between two biological conditions (i.e. AsA-feeding and F-control) including: aspartic acid, cysteine, glutamine, glutamic acid, glycine, leucine, methionine, phenylalanine, proline, galactinol, myo-inositol, citric acid, succinic acid, α-tocopherol, ascorbic acid, phytol, tartaric acid, glycerol, ferulic acid, glyceric acid, phosphoric acid, and urea.</td><td>The abundance of 23 metabolites was significantly different between two biological conditions (i.e. AsA-feeding and F-control) (revised Fig. 5b). These include: cysteine, glutamine, glutamic acid, glycine, leucine, methionine, phenylalanine, proline, threonine, fructose, galactinol, myo-inositol, citric acid, malic acid, α-tocopherol, γ-tocopherol, ascorbic acid, phytol, ferulic acid, GABA, glyceric acid, phosphoric acid, and urea.</td></tr>
<tr><td colspan="2">Metabolites marked as ‘bold’ above are common metabolites that are significantly different between two biological conditions in the original (18 metabolites out of 22) as well as the revised version (18 metabolites out of 23). The trend of metabolic changes of these metabolites is similar in the two versions.</td></tr>
<tr><td colspan="2">Four metabolites (i.e. aspartic acid, succinic acid, tartaric acid, and glycerol) that were significantly higher in AsA-fed leaves compared to F-control in the original version are also higher in AsA-fed leaves than F-control in the revised version but not significantly.</td></tr>
<tr><td colspan="2">Three metabolites (i.e. GABA, malic acid, and γ-tocopherol), significantly higher in AsA-fed leaves compared to F-control in the revised version were also higher in AsA-fed leaves in the original version but not significantly.

Fructose is significantly higher in F-control than AsA-fed leaves in the revised version; however, its abundance in the original version was slightly higher in AsA-fed leaves although not significantly.

Threonine is significantly higher in AsA-fed leaves than F-control in the revised version; however, its abundance in the original version was higher in F-control though not significantly.</td></tr>
</table>

A **revised Supp. Table 1** shows the new concentration data for each metabolite.

Principal component analysis (PCA) was also performed using the new data for the revised version. As in the original version, there was clear separation between the two treatments (**revised Fig. 5a for PCA and revised Supp. Table 2 for the new loading values for each metabolite**).

A slight change in **Fig 6** that we have made is the addition of gamma-tocopherol which was not found to be significant in the original version.

Minor comments:

1. 82 exists.

- We have replaced ‘exits’ with ‘exists’ on line 87.

2. 236 and 237 and table 1: FW/DW and DW/FW are redundant information please stick to one parameter.

- We have removed redundancy of FW or DW data from this table as recommended. See revised Table 1. and the text on line 242 to 245.

3. Fig. 4A looks a bit misleading compared to the other subfigures. Please change the y-axis accordingly. (=linear from 0-3XXX).

- We have **revised ‘y-axis’** in Figure 4A as per reviewer’s suggestion by removing the ‘break’ and making it a ‘linear’ range.

4. ll. 279-282 please shorten.

- Done. See line 284 to 286

5. Fig. Please use larger points.

- We have increased the font size in figures.

6. Figure legends: Replication (n=X) for the production of the p-values is not mentioned here.

- We added information of replication numbers that were used to calculate p-values under section of ‘Figure Legends’. See line 775 to 778 (Figure 2); line 797 to 799 and line 800 to 801 (Figure 3); line 807 (Figure 5).
